# Project Khanya: a randomized, hybrid effectiveness-implementation trial of a peer-delivered behavioral intervention for ART adherence and substance use in Cape Town, South Africa

**DOI:** 10.1186/s43058-020-00004-w

**Published:** 2020-03-04

**Authors:** Jessica F. Magidson, John A. Joska, Bronwyn Myers, Jennifer M. Belus, Kristen S. Regenauer, Lena S. Andersen, Sybil Majokweni, Conall O’Cleirigh, Steven A. Safren

**Affiliations:** 1 Department of Psychology, University of Maryland, 4094 Campus Drive, College Park, MD USA; 2 HIV Mental Health Research Unit, Division of Neuropsychiatry, Department of Psychiatry and Mental Health, University of Cape Town, Cape Town, South Africa; 3 Alcohol, Tobacco and Other Drug Research Unit, South African Medical Research Council, Cape Town, South Africa; 4 Division of Addiction Psychiatry, Department of Psychiatry and Mental Health, University of Cape Town, Cape Town, South Africa; 5 Department of Psychiatry, Massachusetts General Hospital/Harvard Medical School, Boston, USA; 6 Department of Psychology, University of Miami, Coral Gables, USA

**Keywords:** RE-AIM, Hybrid design, Global mental health, HIV, Substance use, Antiretroviral therapy (ART) adherence

## Abstract

**Background:**

Substance use is prevalent in South Africa and associated with poor HIV treatment outcomes, yet, it is largely unaddressed in HIV care. Implementing an evidence-based, task-shared intervention for antiretroviral therapy (ART) adherence and substance use integrated into HIV care may be a feasible and effective way to improve HIV treatment outcomes and reduce substance use in this population.

**Methods:**

Guided by the RE-AIM framework, a randomized, hybrid type 1 effectiveness-implementation trial (*n* = 60) is being used to evaluate a peer-delivered intervention that integrates evidence-based intervention components, including Life-Steps (problem solving and motivational skills for HIV medication adherence), behavioral activation to increase alternative, substance-free rewarding activities in one’s environment, and relapse prevention skills, including mindfulness. The comparison condition is enhanced standard of care, which includes facilitating a referral to a local substance use treatment clinic (Matrix). Participants are followed for a period of 6 months. Implementation outcomes are defined by Proctor’s model for implementation and include mixed methods evaluations of feasibility, acceptability, and fidelity, and barriers and facilitators to implementation. Primary patient-level effectiveness outcomes are ART adherence (Wisepill) and substance use (WHO-ASSIST and urinalysis); viral load is an exploratory outcome.

**Discussion:**

Results of this trial will provide important evidence as to whether peer delivery of an integrated intervention for ART adherence and substance use is feasible, acceptable, and effective. Implementation outcomes will provide important insight into using peers as an implementation strategy to extend task sharing models for behavioral health in resource-limited settings globally.

**Trial registration:**

ClinicalTrials.gov identifier: NCT03529409. Trial registered on May 18, 2018.

Contributions to the literature

- Project Khanya will extend the existing literature by improving our understanding of the implementation of peer-delivered behavioral interventions for substance use and HIV medication adherence in a resource-limited setting with one of the greatest HIV burdens globally.
- Implementation science methods are needed to evaluate task sharing models in global mental health.
- Resource-limited settings with the greatest HIV burden face unique implementation barriers.
- A peer-delivered integrated behavioral intervention such as the one evaluated in Project Khanya may be a feasible, acceptable, efficient, and scalable approach to extending the reach of substance use care for people living with HIV globally.

## Introduction

South Africa (SA) is home to the largest number of people living with HIV (PLWH) in the world—approximately 7.7 million [1]. With limited antiretroviral therapy (ART) regimens available in SA, there is high risk of ART nonadherence and treatment failure [2]. Alongside the HIV epidemic, there has been an alarming increase in substance use (SU) in SA [3, 4], though SU is largely unaddressed in HIV care. This is a “missed opportunity” for maximizing HIV treatment, given that untreated, problematic SU is associated with worse ART adherence, lower rates of viral suppression, and greater likelihood of HIV transmission [5–9]. SU treatment utilization in SA is low; only approximately 2.3% of individuals seeking care receive minimally adequate treatment [10]. Although integrated services are more efficacious [11] and there is a push towards integrated care models for PLWH in low and middle-income countries (LMICs) [12] and in SA specifically [13], this is not a routine practice for ART adherence and SU treatment in SA [14]. Implementation science research is needed to understand how to feasibly, effectively, and efficiently integrate SU treatment into HIV care in SA.

In particular, sustainability must be considered, especially for LMICs. Given the lack of availability of trained mental health and SU professionals in this setting, an implementation strategy will require “task sharing” [15, 16]. Task sharing models include hiring and training less specialized health care workers, such as community health workers (CHWs) or lay counselors, to deliver mental health services under the supervision of more highly trained professionals [17]. Task sharing mental health treatment has largely focused on training lay adherence counselors and CHWs and to a much lesser extent peers [18, 19]. When considering scale-up of task-shared mental health interventions, there are known implementation barriers using lay adherence counselors and/or CHWs, including high caseloads, already being burdened with numerous clinical demands, as well as high levels of stigma towards SU [20]. Peers add an ability to share lived experience that can foster connection with patients and potentially reduce stigma [21]. Indeed, formative work leading up to this trial pointed to an overwhelming preference for peer-delivered SU counseling [20]. Peer-delivered SU interventions have been scaling in the USA and have been shown to be efficacious for improving engagement in care, reducing costly utilization, and improving SU outcomes [22].

This trial builds upon prior and ongoing work in SA that has largely, until recently, focused on efficacy (vs. implementation) research for treating alcohol use among PLWH [14, 23, 24]. The current trial builds upon these prior studies in several ways, including (1) screening individuals who are also using other substances in addition to alcohol; (2) targeting recruitment of individuals who are struggling with ART adherence, engagement in care, and/or viral suppression; (3) using a hybrid effectiveness-implementation design; and (4) utilizing an implementation strategy of peer delivery as opposed to an HIV counselor or CHW.

### Trial objectives

The primary objective of this trial is to evaluate the effectiveness and implementation of an integrated, peer-delivered SU and ART adherence intervention delivered in HIV care in SA. This trial will contribute important insight into the implementation strategy of using peers in a task sharing model—specifically whether a peer-delivered behavioral intervention for SU in HIV care will be feasible, acceptable, and delivered with fidelity and how this compares with prior evidence using other lay health worker task sharing models.

## Methods

This manuscript is in accordance with the Standard Protocol Items: Recommended items to address in a clinical trial protocol and related documents (SPIRIT) guidelines. See Additional file 1 SPIRIT checklist.

### Trial design

This study is using a hybrid type 1 effectiveness-implementation design [25], guided by RE-AIM [26, 27], given its inclusion of patient- and provider-level outcomes and prior application in sub-Saharan Africa [28, 29]. The intervention being evaluated is a combination of efficacious treatment components, but because it had not previously been delivered in this setting, we elected to use a hybrid type 1 design; however, a primary emphasis of this trial is on the implementation in this new context. Implementation outcomes were defined based upon Proctor’s model and recommendations for early implementation assessments [30]. See Table 1 for a description of the RE-AIM constructs and implementation outcome measurements in the current trial. This study is a randomized controlled trial (RCT) with two parallel arms: enhanced standard of care (ESOC) and the active behavioral intervention (locally named “Khanya”), which includes a combination of efficacious intervention components locally adapted for this setting (see descriptions below).

**Table 1:** RE-AIM framework applied to study outcomes

Dimension	Description	Outcome assessment
Reach	Intervention ability to reach participants who have both SU problems and ART nonadherence	• % of patients screened who meet eligibility criteria for study enrollment
Effectiveness	ART adherence (primary)	• Number of doses missed divided by the number of prescribed doses (measured via Wisepill)
	SU (primary)	• Urinalysis (yes/no) and self-reported total score on the WHO-ASSIST score
	Viral suppression (exploratory)	• Viral load copies per ml of blood
Adoption	Provider and organization perceptions of feasibility/acceptability and future uptake	• Qualitative interviews with providers and organizational leadership based on RE-AIM to assess perceptions and likelihood of uptake following the trial; longer-term adoption to be assessed in the subsequent trial following from this pilot study
Implementation	Feasibility	• Pragmatic implementation science measure based on RE-AIM (14-item feasibility subscale) [31] • % assigned to the intervention who agree to enroll • Qualitative interviews with patients structured based on RE-AIM
	Acceptability	• Pragmatic implementation science measure based on RE-AIM (15-item acceptability subscale) [31] • Retention: % attending ≥ 1 session, % attending ≥ 75% of sessions, uptake of booster sessions, and % who dropped out of the intervention • Qualitative interviews with patients structured based on RE-AIM
	Intervention fidelity	• Independent rater and interventionist self-report on randomly selected 20% of intervention sessions
Maintenance	Provider continued usage of Khanya to treat co-occurring SU and ART nonadherence	• To be evaluated in the subsequent trial following from this pilot study

### Participants and study procedures

**Fig. 1:**
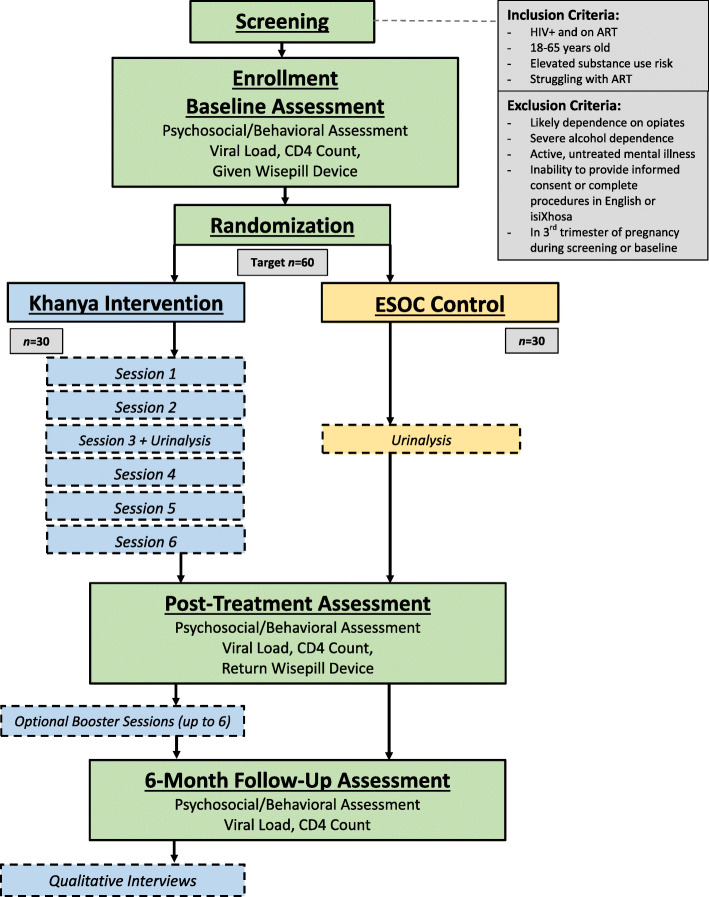
CONSORT diagram

The study flow CONSORT diagram is illustrated in Fig. 1. Patients are recruited from an HIV clinic in Khayelitsha, a peri-urban area of Cape Town that is predominantly Black African and isiXhosa speaking with the highest HIV prevalence in SA’s Western Cape Province [32]. Potentially eligible and interested patients are approached by study staff for screening. All patients are informed that study participation will in no way affect their treatment at the clinic. Screened participants who meet study inclusion criteria (see below) complete informed consent processes prior to baseline by a trained study research assistant, including a release of relevant medical information, and then complete a baseline assessment. The baseline assessment includes a battery of measures related to HIV infection history, medication adherence, and current and past SU. Participants also begin monitoring their ART adherence using Wisepill [33], a real-time, wireless electronic adherence monitoring device. Randomization to intervention condition occurs 2 weeks after the baseline assessment to establish a baseline level of ART adherence. At randomization, participants are randomly assigned (1:1) to either ESOC or Khanya using a computer-generated chart in the randomization module on REDCap [34]. The study staff clicks “randomize” in REDCap, and the participant’s condition is revealed and cannot be changed. Participants then complete the following major assessment time points: mid-point, post-treatment, and 6-month follow-up. See Additional file 2 for the schedule of enrollment, assessments, and interventions. The post-randomization assessments are conducted by a research assistant who is blinded to participant condition^1^ with no a priori circumstance under which unblinding is permitted. All participants receive ZAR 150 (~ $10 USD) grocery voucher for major assessments. Participants do not receive financial incentives to attend therapy sessions beyond reimbursing travel costs incurred in order to attend therapy visits (ZAR 50; ~$3 USD).

#### Eligibility criteria

Eligibility criteria include (1) HIV positive and on ART; (2) 18–65 years of age; (3) moderate SU measured by the WHO Alcohol, Smoking, and Substance Involvement Screening Test (WHO-ASSIST) [35] (alcohol ≥ 11 or other SU ≥ 4, indicating moderate risk of health and other problems); and (4) ART nonadherence in the past 3 months, as measured by either (a) missing at least one refill of ART, (b) reinitiating first-line treatment, (c) being on second-line treatment, or (d) having a detectable viral load. Exclusion criteria are (1) severe opiate use (WHO-ASSIST score > 26) given that opiate substitution therapy may not be available and a higher level of care may be needed, (2) severe alcohol dependence symptoms that may warrant medical management of withdrawal symptoms, (3) active uncontrolled major mental illness (e.g., psychosis, mania) that interferes with study participation, (4) inability to provide informed consent and/or complete procedures in English or isiXhosa, (5) in the third trimester of pregnancy during screening or baseline, or (6) currently enrolled in Matrix or another SU treatment program or study focused on ART adherence or SU.

### Interventions

#### ESOC

Given the implementation science focus, we elected to use a version of standard of care (SOC) as our study comparator. SOC for the treatment of SU, if and when detected in HIV care in SA, is a referral to a local SU treatment clinic. In Khayelitsha, there is a city-funded SU treatment program co-located at the HIV care study site, which follows the Matrix model [36, 37], an evidence-based 16-week program to treat SU. Although the two sites are not connected from the inside, a Matrix treatment center shares a driveway and a wall with the clinic at which this study is taking place. Yet, there is evidence from our preliminary work from this study that clinic patients may not be aware of Matrix [20]. Therefore, we enhance SOC (ESOC) by facilitating screening for SU using the WHO-ASSIST and support the referral to Matrix, which includes offering to accompany participants to the Matrix clinic to schedule an intake, providing a direct referral, and following up on the referral at each subsequent study visit.

#### Khanya intervention

The Khanya intervention is a six-session, peer-delivered intervention that includes a combination of efficacious intervention components for ART adherence and SU, including Life-Steps (problem solving and motivational skills for HIV medication adherence) [38], motivational interviewing (MI) to explore readiness to change SU and adherence behaviors, behavioral activation to increase alternative, substance-free rewarding activities in one’s environment [39, 40], and relapse prevention skills, including mindfulness [41, 42]. Formative qualitative work [20] was used to adapt the intervention content and implementation strategy to ensure the relevance of the efficacious intervention concepts (e.g., mindfulness, values) to the local culture and to promote feasibility and acceptability of the approach. When participants present for each intervention session, a graph of their ART adherence from the Wisepill device is printed from the server prior to the session and used to aid the discussion of challenges to ART adherence. Participant SU is assessed by a research assistant (not the interventionist) using the Timeline Follow-Back (TLFB) prior to the beginning of each session. The TLFB is used to guide the review of SU in-session and discussed together using an MI approach to explore patterns of use in line with the participant’s goal around SU; this continues in each session as the participant learns skills to address SU, its interference with ART adherence, and other substance-related problems. Each session lasts approximately 45 min to 1 h and builds upon prior session content. Each session ends with a brief mindfulness exercise, and participants are also given a CD with audio recordings of the brief mindfulness exercises in isiXhosa that are covered at the end of each session. Home practice of the intervention skills is assigned between sessions and reviewed at each subsequent session. All sessions are audio recorded for fidelity monitoring. Participants are also offered up to six optional booster sessions that focus on continued practice of skills and relapse prevention. See our team’s prior research [43–45] for greater detail on the intervention components.

### Implementation strategies

**Fig. 2:**
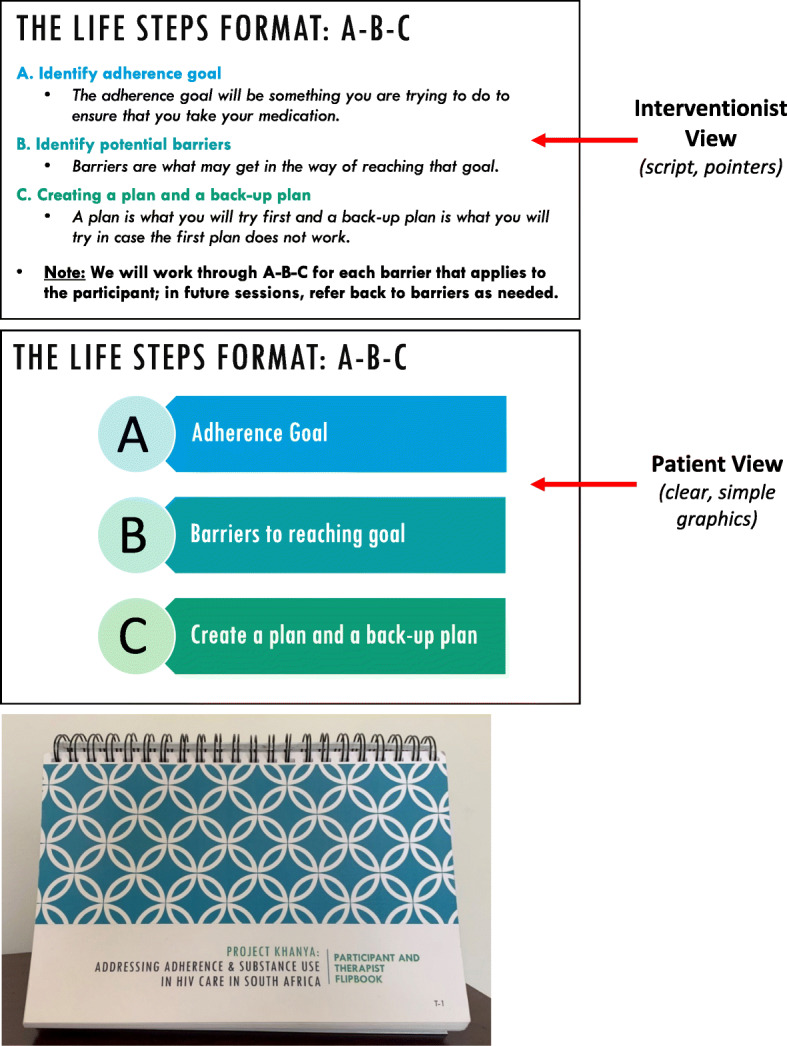
Depiction of flipchart implementation strategy to promote fidelity in task sharing model

Two implementation strategies that emerged from our formative work included the need for (1) peer delivery of the intervention (i.e., a lay counselor with lived experience with SU and/or HIV) and (2) the use of a flipchart to promote fidelity [20]. We tailored the approach for peer delivery by building flexibility into our intervention, training, and supervision procedures to allow for self-disclosure to accommodate the unique perspective of a peer interventionist and adapted our fidelity assessment to also include these elements. Regarding the flipchart, the ultimate aim is to maximize fidelity while also enhancing participant engagement and comprehension (see Fig. 2). While the participant sees mostly visual depictions of the concepts, the interventionist has a guide on the content to deliver and is able to maintain eye contact while following the structured guide for content delivery.

### Peer interventionist training

Interventionist training was led by a US-based clinical psychologist (JFM) in SA over 5 days. The training was guided by the flipchart and included a combination of didactic training, interactive, experiential learning of intervention components, including developing one’s own mindfulness practice, and role plays. Following the 5-day in person training, ongoing training and supervision was conducted weekly via videoconferencing and included reflections on one’s own mindfulness practice and personal experience with behavioral activation and feedback on role played sessions. Role plays of the full intervention were conducted with five mock participants and video recorded to allow for ongoing training and fidelity monitoring to determine readiness to begin delivering the intervention.

### Supervision

The interventionist receives 1 h of weekly supervision from a US-based PhD-level psychologist with expertise in behavioral interventions for medication adherence and substance use. Supervision sessions are held over Webex, a secure video teleconference service. The interventionist translates one session per week from isiXhosa to English for the supervisor to listen to and provide feedback (separate from the randomly selected sessions that are translated for fidelity ratings). Supervision is focused on fidelity, non-specific clinical skills of the interventionist (e.g., being supportive and nonjudgmental, appropriate self-disclosure), time management, and strategies to reduce burnout.

### Study measures

#### Implementation outcomes

Guided by RE-AIM [26, 27] and Proctor’s model [30], we are assessing early implementation outcomes, including a mixed methods evaluation [46] of acceptability and feasibility, and fidelity of peer delivery.

*Acceptability*, defined as the tolerability or satisfaction of the approach [30], is assessed using two quantitative assessment measures: (1) a 15-item subscale of a pragmatic, quantitative assessment based on RE-AIM [31] developed by the Applied Mental Health Research group (AMHR) at Johns Hopkins University [31] and (2) quantitative metrics of patient attendance, uptake, and retention in the intervention. The AMHR pragmatic, quantitative assessment [31] is a validated measure assessing key implementation domains for a mental health intervention in a LMIC. Sample items on the acceptability subscale include “Did you feel satisfied with the program’s services?” and “Did you enjoy learning skills from the program?” Psychometric properties of the scale show good internal consistency (∝ = .89) and test-retest reliability (rho = .70).

*Feasibility*, defined as the fit or utility of the intervention or suitability and practicability for this setting [30], is also being assessed using two quantitative assessments: (1) the 14-item feasibility subscale of the AMHR pragmatic assessment measure based on RE-AIM [31] and (2) the percentage assigned to the intervention who agree to enroll in the intervention. The feasibility subscale also shows good psychometric properties (internal consistency ∝ = .85; test-retest reliability rho = .79). Sample items from the feasibility subscale include “Have you been able to attend the sessions of the program without difficulty?” and “Did you have enough money to pay for transport to get to the program sessions?” Secondly, we will compare the rates of patient enrollment and participation across conditions with other similar pilot trials [47, 48].

To supplement the quantitative assessments of feasibility and acceptability, qualitative interviews with up to 30 participants are also assessing perceptions of acceptability and feasibility of the intervention, as well as barriers and facilitators to implementation. Qualitative interview guides are based upon Proctor’s model for implementation outcomes [30, 49] and the RE-AIM framework [26, 27]. We are using the qualitative interviews to gain more specificity on which elements of the intervention are acceptable and feasible, as well as elicit participant feedback on ways to improve these implementation outcomes going forward.

*Fidelity* is being assessed two ways: independent rater and interventionist self-report. The independent rater is listening to a randomly selected subset (20%) of intervention sessions that are translated from isiXhosa to English. A 15- to 19-item fidelity assessment was developed for each session that includes an assessment of whether each core intervention content component was delivered. At the end of each session, the interventionist completes an identical self-report form assessing her level of fidelity to the intervention. In addition to rating content, the independent rater assesses sessions for (a) appropriate self-disclosure given the peer interventionist implementation strategy; (b) presence of actively stigmatizing behaviors; and (c) therapeutic common factors including exhibiting warmth and non-judgmental attitudes, which were adapted from prior research on cross-cultural common factors [50].

#### Effectiveness outcomes

*ART adherence* is assessed using Wisepill, a real-time, wireless electronic adherence monitoring device previously used in clinical research in sub-Saharan Africa to assess ART adherence [33]. Wisepill uses cell phone technology to transmit a real-time signal to a web server when the pill box is opened. Participants do not need to come into the clinic for the study to obtain readings. A dose is counted as “taken” if the box is opened ± 2 h from the prescribed time. As has been conducted in prior adherence research (e.g., [51, 52]), adherence will be measured as the number of doses missed divided by doses prescribed between randomization and the post-treatment assessment. The 2-week period from baseline to randomization is the baseline adherence measure.

*SU* is assessed via both a biological measure (urinalysis) and self-report. Urinalysis is conducted at all-time points using the Confirm BioSciences rapid detect 6-panel urine tests (cocaine, marijuana, amphetamines, opiates, phencyclidine, and alcohol). Urinalysis is supplemented with mandrax testing (a commonly used sedative in the area) using the Methaqualone urine rapid test stick. The WHO-ASSIST, a self-report assessment of SU (alcohol, cannabis, cocaine, opiates, amphetamines, hallucinogens, and other drugs), is administered at screening, when substance use risk in the past 3 months is assessed. At the post-treatment and the follow-up assessments, alcohol and drug use are assessed since the prior assessment. It categorizes individuals into low (0–3 for illicit drugs/0–10 for alcohol), moderate (4–26 for illicit drugs/11–26 for alcohol), or high risk (> 26) for substance use-related problems. The use of the WHO-ASSIST in primary care has been validated in SA [35]. To supplement the WHO-ASSIST, we also administer the TLFB to assess the frequency of alcohol and/or drug use in the past 2 weeks at each assessment. To promote accurate recall of the quantity of alcohol use on the TLFB, participants are aided by the use of empty, locally recognizable alcohol containers that reflect the commonly used forms of alcohol in this population.

*Viral suppression*, which is measured as the number of HIV viral copies per ml of blood, is extracted from participants’ medical record at each assessement. If viral load results are not available within 3 months of the baseline assessment or 30 days of the two follow-up assessments, participants undergo a separate blood draw for viral load assay at no cost to the patient. Blood samples are stored at the University of Cape Town and tested by the National Health Laboratory Service.

### Power considerations and sample size calculations

The main analysis on which the sample size calculation for the quantitative analysis was based is the effect of the experimental intervention vs. ESOC on ART nonadherence at post-treatment. Previous objective measures of ART adherence in substance using populations have estimated average adherence rates of approximately 55% with a standard deviation of 25% [53–55]. Assuming similar characteristics in ESOC and based on a sample size of 30 in each arm (recommended as the upper limit sample size for pilot RCTs [56] and pilot hybrid type 1 effectiveness-implementation designs [25, 57]), we will have 80% power to detect an 18% difference in objectively measured ART adherence between study arms using a two-sided test with an alpha level of .05. For qualitative analyses, as recommended in qualitative research [58], proposed sample sizes are estimates of the number of interviews needed to reach theoretical saturation.

### Data management and data analysis

Quantitative data are collected using the REDCap interface on a tablet. Data are reviewed twice for quality assurance including checking consistency of participant responses and ensuring that reported values fall within the range of possible values. TLFB is administered using a hardcopy calendar to allow for visual aids to guide participant recall, and these data are later entered into REDCap and double checked for accuracy. Wisepill data are stored on the Wisepill server. Wisepill devices are checked weekly on the server to ensure devices are operational.

Descriptive statistics will be generated to evaluate implementation outcomes. Total scores from the acceptability and feasibility subscales of the AMHR pragmatic, implementation assessment [31] will be created; these scores, alongside data obtained on provider fidelity to the intervention and patient participation and retention, will first be evaluated descriptively. The means and standard deviation of provider fidelity at each session will be calculated. Average session fidelity will be compared with similar lay counselor delivered interventions implemented in SA and peer-delivered interventions in the US; similarly, the percentage of patients participating and retained in the intervention will be described and compared with similar pilot studies [23, 59].

Data from individual interviews to assess implementation outcomes will be analyzed iteratively and follow descriptive qualitative thematic analysis [60]. Guided by RE-AIM and Proctor’s model for defining implementation outcomes, initial concepts related to the central research questions listed will be identified based on the semi-structured interview guide. Concepts will be used to develop a codebook consisting of a label, a definition, and illustrative quotes from the data. All transcripts will be coded independently by two research assistants. Final themes will be agreed upon and inter-rater reliability of coding assessed.

Following recommendations of mixed methods designs [46], we will integrate findings from the qualitative and quantitative methods using a convergent QUANT + qual mixed methods design to assess implementation outcomes [46]. Specifically, themes from the qualitative interviews will be compared with the quantitative descriptive data on feasibility and acceptability, assessing for both convergence and divergence.

Quantitative longitudinal analysis will examine changes in ART nonadherence, SU, and viral load from baseline to post-treatment between the conditions. If appropriate after examining descriptive data, normality, and assumptions, *t* tests may be used to compare continuous variables (e.g., age, ART nonadherence) and Fisher’s exact test may be used to compare categorical variables (e.g., gender, urinalysis). If the appropriate statistical assumptions are met after examining distributions of main outcome variables, longitudinal data analysis using generalized linear mixed models (GLMM) may be used to compare the two study arms over time. This approach increases power by including all available data points. We will adjust the analyses for relevant baseline demographic characteristics (e.g., gender, age) for each analysis and clinical differences that exist between the two treatment arms. Intent-to-treat analyses will be utilized, where all individuals will be analyzed according to the condition to which they were randomized.

### Ethical considerations and trial management

This study is approved by the University of Cape Town (UCT) Human Research Ethics Committee (HREC). Data are de-identified by using participant ID numbers (not names) on all study materials. The document linking participant identifiable information (e.g., names, phone numbers) is stored on a secure institutional server, password protected, and only accessible to the study staff who need access to this information to perform the study duties.

This study has oversight by a Data Safety and Monitoring Board (DSMB). The DSMB meets annually to review data collection procedures and ensure the scientific and ethical rigor of the study is maintained. The DSMB for this trial is comprised of three leading scientists who have expertise in behavioral interventions, HIV, substance use, implementation science, and SA. The DSMB members are independent of the sponsor without competing interests for providing rigorous scientific and ethical oversight. There are no a priori stopping rules for discontinuing or modifying provision of the experimental intervention, although our DSMB has this right and responsibility.

We collect information on adverse events that are experienced by enrolled participants. Adverse events are classified serious/non-serious, related/unrelated to the study intervention, and expected/unexpected. All serious adverse events (i.e., any event considered to be fatal, immediately life-threatening, permanently or substantially disabling, requiring or prolonging inpatient hospitalization, or any congenital anomaly) are reported to UCT HREC within 24 h. We report all adverse events to our DSMB committee yearly.

Key ethical considerations in this trial include protecting the confidentiality and well-being of PLWH and SU. We closely monitor and screen for medically complicated withdrawal symptoms for patients with severe alcohol use who are reducing their alcohol intake as part of the intervention. We maintain a close relationship with the co-located Matrix substance use treatment facility and the medical officer at the clinic to ensure patient safety throughout the trial.

All protocol modifications are submitted as ethics amendments to the UCT HREC. Any major protocol modifications will be reflected on ClinicalTrials.gov. Participants are re-consented following any protocol modifications that would impact their participation in the study, and the UCT HREC approves whether it is necessary to re-consent patients if a major protocol amendment is requested.

## Discussion

Given the prevalence of SU among PLWH in SA and interference with HIV care outcomes across the continuum [5–7, 9, 61], it is crucial to identify how to integrate and implement evidence-based interventions to treat substance use in HIV care in SA. Identifying intervention components that can be feasibly and sustainably delivered using a task sharing model is essential, as there are significant workforce shortages for behavioral health interventions in SA and other LMICs [15, 62]. Task sharing efforts in SA and elsewhere to date have largely involved task sharing with lay counselors, such as lay adherence counselors or CHWs, as opposed to peers [15, 16, 18, 19]. This trial will contribute new knowledge about the effectiveness and implementation of a peer-delivered intervention using a task sharing model. This study will allow us to gain understanding regarding the feasibility, acceptability, fidelity, and effectiveness of this peer-delivered approach and how these implementation and effectiveness outcomes compare with other lay health worker-delivered task-shared substance use interventions [18, 19]. An integrated intervention that simultaneously addresses substance use and ART adherence offers a parsimonious approach if feasible for peer delivery. As the SA Department of Health refines their strategy for integration of behavioral health services into primary health care facilities, findings may have important implications to understand what may be effective and feasible in this context.

Potential challenges in this trial include difficulties with recruiting and retaining patients who are struggling with SU, ART nonadherence, and engagement in care. By prioritizing recruitment of individuals who have already demonstrated challenges with engagement in care, we are more likely to recruit individuals who will have greater challenges also in engaging in study-related appointments and are difficult to retain. Further, to date, approximately 40% of participants have not owned a cell phone, which makes tracking and retaining participants more challenging. Starting from the point of screening through the 6-month follow-up assessment, retaining this hard-to-reach population is an important priority in this trial, while also balancing real-world implementation that may have limited resources to track and retain difficult populations. Efforts to address these anticipated challenges include identifying participants who we are struggling to retain during weekly team supervision and collecting contact numbers for two people who are likely to know the whereabouts of the participant.

### Design considerations and future directions

There are numerous design considerations and decisions that reflect our dual focus on both effectiveness and implementation. For instance, we elected to include booster sessions in our trial design based upon feedback from participants and interventionist observation that additional skill practice would be needed to sustain behavior change, especially in light of fluctuating motivation. However, we also recognize the need to prioritize brevity in our intervention design to ultimately have an intervention package that will enhance the probability of policy uptake within SA’s Department of Health. Thus, we elected to offer optional booster sessions with no financial incentives to provide the option to reinforce ongoing skill practice and relapse prevention and evaluate uptake of these optional booster sessions as an additional measure of acceptability.

Primary limitations relate to this being a pilot trial, including a small sample size and relatively short-term follow-up period. There are also limitations when considering future scale-up of the intervention, including reimbursement for transport costs and financial incentives provided for participating in research assessments and the fact the interventionist was hired to deliver the intervention and was not balancing other clinic responsibilities. This trial is a first step towards evaluating whether this intervention has potential for future scale-up. If it proves to be effective in this setting and early implementation outcomes are promising, next steps would require a larger trial to evaluate longer-term effectiveness and implementation outcomes, including cost effectiveness, maintenance, and sustainability. Further, these outcomes must be assessed at the provider and organizational levels using a greater number of clinic sites and peer interventionists in a future cluster randomized clinical trial. Ultimately, our aim is to develop and evaluate an intervention and implementation strategy that can be scaled up in SA and other LMICs to expand access to evidence-based HIV and behavioral health care in resource-limited primary care settings.

### Dissemination plan

The results of this study will be published after all data is collected, cleaned, and analyzed, and the results will be updated in ClinicalTrials.gov within 1 year of the primary completion date. We will also update NIH on the study results in annual progress reports to NIDA. Additionally, we will report back on the study results to the City of Cape Town Health Department, including by holding forums for HIV and SU treatment to share results with clinical providers, patients, and staff.

## Supplementary information

**Additional file 1.** SPIRIT Checklist.

**Additional file 2.** Schedule of enrollment, intervention, and assessments.

## Data Availability

Data sharing is not applicable to this article as no datasets were generated or analyzed during the current study. However, we will grant public access to the full protocol, de-identified participant-level dataset, and statistical code for interested parties by emailing the study PI. All study key personnel will have access to the final de-identified trial dataset without restrictions.

## Abbreviations

ART: Antiretroviral therapy
CHW: Community health worker
DSMB: Data Safety and Monitoring Board
ESOC: Enhanced standard of care
GLMM: General linear mixed models
HREC: Human Research Ethics Committee
IAA: Independent Authorization Agreement
IEC: Independent Ethics Committee
IRB: Institutional Review Board
LMIC: Low and middle-income country
MI: Motivational interviewing
NIMH: National Institute of Mental Health
PLWH: People living with HIV
PS: Problem solving
RCT: Randomized controlled trial
SA: South Africa
SOC: Standard of care
SU: Substance use
TLFB: Timeline Follow-Back
UCT: University of Cape Town

## Footnotes

1 Blinded assessor was introduced after ten participants completed the post-treatment assessment and one participant completed the 6-month follow-up due to staffing constraints at study initiation.
