# Supplementary material for: Project Khanya: a randomized, hybrid effectiveness-implementation trial of a peer-delivered behavioral intervention for ART adherence and substance use in Cape Town, South Africa
Source: Implement Sci Commun. 2020 Mar 4;1:23. doi: 10.1186/s43058-020-00004-w (PMC7326344; doi:10.1186/s43058-020-00004-w)
Supplement: Supplementary file 2 — Additional file 2. Schedule of enrollment, intervention, and assessments. [file 43058_2020_4_MOESM2_ESM.docx]

Table 2. Schedule of enrollment, intervention, and assessments.

|  | **Study Period** | | | | | |
| --- | --- | --- | --- | --- | --- | --- |
|  | **Screening** | **Baseline** | **Randomization** | **Midpoint** | **Post-treatment** | **Follow-up** |
| **TIMEPOINT**** | **< 12 weeks before baseline** | **2 weeks before randomization** | **0** | ***4 weeks*** | ***10 weeks*** | ***22 weeks*** |
| **ENROLLMENT:** |  |  |  |  |  |  |
| ***Eligibility screen*** | X |  |  |  |  |  |
| ***Verbal informed consent*** | X |  |  |  |  |  |
| ***Written informed consent*** |  | X |  |  |  |  |
| ***Allocation*** |  |  | X |  |  |  |
| **INTERVENTIONS:** |  |  |  |  |  |  |
| ***Khanya*** |  |  |  |  |  |  |
| ***Enhanced Standard of Care*** |  |  |  |  |  |  |
| **ASSESSMENTS:** |  |  |  |  |  |  |
| **IMPLEMENTATION** |  |  |  |  |  |  |
| ***RE-AIM feasibility/***  ***acceptability quantitative assessment***^45^ |  |  |  |  | X |  |
| ***Qualitative assessment of feasibility and acceptability guided by Proctor model***^30^ |  |  |  |  |  | X |
| ***Fidelity***** |  |  | X | X | X | X |
| **EFFECTIVENESS** |  |  |  |  |  |  |
| ***Wisepill**** |  | X | X | X | X | X |
| ***Urinalysis*** |  | X |  | X | X | X |
| ***WHO ASSIST*** |  | X |  |  | X | X |
| ***TLFB***** |  | X |  | X | X | X |
| ***Viral load test*** |  | X |  |  | X | X |

*Wisepill is continuously measured between baseline and post-treatment assessment.

**Participants randomized to Khanya have this assessment completed at each intervention session and 20% of sessions are rated by an independent assessor.
